# Fold-Change-Specific Enrichment Analysis (FSEA): Quantification of Transcriptional Response Magnitude for Functional Gene Groups

**DOI:** 10.3390/genes11040434

**Published:** 2020-04-17

**Authors:** Daniil S. Wiebe, Nadezhda A. Omelyanchuk, Aleksei M. Mukhin, Ivo Grosse, Sergey A. Lashin, Elena V. Zemlyanskaya, Victoria V. Mironova

**Affiliations:** 1 Institute of Cytology and Genetics Siberian Branch of the Russian Academy of Sciences (SB RAS), 630090 Novosibirsk, Russia; daniil.wiebe@gmail.com (D.S.W.);; 2 Institute of Computer Science, Martin Luther University Halle-Wittenberg, 06120 Halle, Germany; 3 German Centre for Integrative Biodiversity Research (iDiv) Halle-Jena-Leipzig, 04103 Leipzig, Germany; 4 LCT & EB, Faculty of Natural Sciences, Novosibirsk State University, 630090 Novosibirsk, Russia

**Keywords:** gene expression, gene ontology, enrichment analysis, transcriptomics

## Abstract

Gene expression profiling data contains more information than is routinely extracted with standard approaches. Here we present Fold-Change-Specific Enrichment Analysis (FSEA), a new method for functional annotation of differentially expressed genes from transcriptome data with respect to their fold changes. FSEA identifies Gene Ontology (GO) terms, which are shared by the group of genes with a similar magnitude of response, and assesses these changes. GO terms found by FSEA are fold-change-specifically (e.g., weakly, moderately, or strongly) affected by a stimulus under investigation. We demonstrate that many responses to abiotic factors, mutations, treatments, and diseases occur in a fold-change-specific manner. FSEA analyses suggest that there are two prevailing responses of functionally-related gene groups, either weak or strong. Notably, some of the fold-change-specific GO terms are invisible by classical algorithms for functional gene enrichment, Singular Enrichment Analysis (SEA), and Gene Set Enrichment Analysis (GSEA). These are GO terms not enriched compared to the genome background but strictly regulated by a factor within specific fold-change intervals. FSEA analysis of a cancer-related transcriptome suggested that the gene groups with a tightly coordinated response can be the valuable source to search for possible regulators, markers, and therapeutic targets in oncogenic processes. Availability and Implementation: FSEA is implemented as the FoldGO Bioconductor R package and a web-server.

## 1. Introduction

Next-generation sequencing technologies revolutionized the field of molecular genetics, providing whole-genome expression profiles for every aspect of life. However, with current analytical tools, we retrieve just a small portion of information encoded in the expression profiles, and the development of new methods is more relevant than ever.

The typical scenario of transcriptome data analysis is the identification of Differentially Expressed Genes (DEGs), followed by a functional enrichment analysis of the gene set using Gene Ontology (GO) [1]. For this, three classes of algorithms are used: Singular Enrichment Analysis (SEA) [2], Gene Set Enrichment Analysis (GSEA) [3], and Modular Enrichment Analysis (MEA) [2]. As a result, GO terms for biological processes, molecular functions, and cellular components associated with the gene expression changes are identified. SEA and MEA identify which functionally-related groups of the genes are overrepresented in differentially expressed genes compared to the genome background; GSEA does that on the entire transcriptome ranking the genes by the fold-change value. Dozens of tools and web-servers exist implementing these algorithms for functional annotation (reviewed in [2,4]).

The limitation of these methods is that they do not give an idea to what extent functionally related genes are coordinated in their expression fold changes. In other words, what is the strength of response for a particular process? To understand the impact of a process in response to a specific factor, it is essential to know if it is weakly, moderately, or highly activated/inhibited. SEA and MEA ignore the fold-change values, GSEA uses them only for ranking the genes. Here we suggest the Fold-change-Specific Enrichment Analysis (FSEA), which identifies functionally-related gene groups that change their expression with a certain strength—fold-change-specifically.

Earlier, we proposed the concept to study the magnitude of response in functionally-related gene groups and showed that there are many GO terms that coordinatively, with certain strengths, were activated or repressed in response to phytohormone auxin in the model plant species *Arabidopsis thaliana* [5]. For example, auxin most strongly upregulates its signaling pathway but it is not the only positive response, as many processes were upregulated moderately (e.g., “histone modifications”) and weakly (e.g., “translation” and “gene expression” in general). Sharma and co-authors found many GO terms that fold-change-specifically differ between drought-tolerant and drought-sensitive rice varieties [6]. Fold-change-specific GO terms were occasionally detected in animal transcriptomes as well, e.g., very weak but significant activation of immunity-related processes have been shown in [7]. However, the role of fold-change-specific transcriptional response has not been studied systematically, because there were no ready-to-use tools.

Here we provide FSEA formal description, adjust and validate the statistical procedures, and implement it as a FoldGO R package and a web-server (https://webfsgor.sysbio.cytogen.ru/). FoldGO application on a cancer-related transcriptome demonstrates that both FSEA/SEA or FSEA/GSEA algorithms should be applied to understand better biological processes underlying cancerogenesis and beyond.

## 2. Materials and Methods 

### 2.1. Datasets for FSEA Testing and Application

#### 2.1.1. Real Datasets for FSEA Application

We used 30 randomly chosen microarray and RNA-Seq datasets from the GEO database [8] in *Homo sapiens* and *Arabidopsis thaliana* (Table S1) on different treatments, conditions, and mutants. Here, as an example, we discuss FSEA the results for one of the datasets, RNA-Seq experiment GSE70466, on the comparison of gene expression between a primary prostate epithelial cell line (HPrEC) and a prostate adenocarcinoma cell line (LNCaP). Starting with raw data, RNA-Seq reads were mapped on the reference *Homo sapiens* genome assembly GRCh38 using STAR aligner [9] followed by quantification with the Rsubread package [10]. Differential expression analysis was conducted using the edgeR package [11].

Oncogenes identification was performed via The Network of Cancer Genes (NCG) database (http://ncg.kcl.ac.uk/query.php) [12].

#### 2.1.2. Simulated Datasets for FSEA Testing

False-positive. To estimate the proportion of false-positive FSEA results, we shuffled two of the real datasets (GSE71334 and GSE70466). To disrupt the relations between the GO terms and the fold change values, the gene identifiers were shuffled 2000 times. FSEA was applied to each of the 2000 shuffled datasets. Then we calculated the proportion of datasets in which at least one fold-specific GO term passed the FDR threshold of 0.05. 

Sensitivity assessment. To simulate the datasets with a specific correlation structure of the fold changes, we created the multidimensional normal distribution, which consists of the following groups:

- Six groups of DEGs of different sizes (5, 10, 20, 30, 40, and 50 genes) with the fold-change mean equals to 1 (μ = 1) and with the strong correlation within each group (correlation coefficient (ρ) > 0.7). These gene sets simulate pseudo-GO terms;
- One group of DEGs (μ = 1) of 100 genes without any correlation (ρ ~ 0);
- One group of non-DEGs (μ = 0) of 700 genes without any correlation (ρ ~ 0).

Then 100 gene sets were sampled from the obtained multidimensional normal distribution.

### 2.2. FSEA Method Formal Description

As an input data, the FSEA method uses a set of genes $G=G_{1},\ldots \ldots ,G_{n}$ and corresponding absolute logarithmic values of fold changes (logFC)$\;X=X_{1},\ldots \ldots ,X_{n}$. The initial set of genes is sorted by the Fold Change (FC) values $G_{\left(1\right)},\ldots \ldots ,G_{\left(n\right)}$, so that $X_{\left(1\right)}<X_{\left(2\right)}<\ldots <X_{\left(n\right)}|X_{\left(1\right)}=\min\left(X_{1},\ldots \ldots ,X_{n}\right),\;X_{\left(n\right)}=\max\left(X_{1},\ldots \ldots ,X_{n}\right)$. Further, the sorted set of genes ***G*** is divided into ***k*** quantiles $\;Q_{1},\ldots \ldots ,Q_{k}$, so that for each $Q_{i}=G_{i,\;1},\ldots \ldots ,G_{i,\;m}$ the following conditions are satisfied:

1. $X_{i,j}<f\left(\frac{i}{k}\right)$;
2. $X_{i,j}\geq f\left(\frac{i-1}{k}\right)$, in case of i>1;
3. $X_{i,j}\geq \min\left(X_{1},\ldots \ldots ,X_{n}\right)$, in case of i=1,

where $X_{i,j}$ is the fold change value for $G_{i,j}|i\in \left\{1,\ldots ,k\right\},\;j\in \left\{1,\ldots ,m\right\}$, and ***f*** is a function, which takes a fraction of the FC values below the boundary of the corresponding quantile as an argument, and returns the FC value corresponding to the boundary of the quantile. Next, $\overset{k}{\underset{n=2}{\sum }}n$ variants for the combinations of neighboring quantiles $\cup_{n=i}^{j}Q_{n}$ are generated, where  i,j∈{1,…,k}, i<j, i≠1 ∧ j≠k.

Further, for each GO term from a preliminarily prepared set $GO=GO_{1},\ldots \ldots ,GO_{s}$, where $GO_{i}=\{G_{i,1},\ldots \ldots ,G_{i,t}\}|\forall \;G_{i,j}\in G,\;i\in \left\{1,\;\ldots ,s\right\},\;j\in \left\{1,\ldots ,t\right\}$ (*t* is the number of genes annotated to the *GO_i_* ) and all quantiles and unions of neighboring quantiles, the enrichment is estimated using Fisher’s exact test for the contingency table (Table 1).

In Table 1, $A=\left|GO_{i}\cap Q_{r}\right|$, $B=\left|GO_{i}\backslash Q_{r}\right|$, $C=\left|Q_{r}\backslash GO_{i}\right|$, $D=\left|G\backslash (Q_{r}\cup GO_{i)}\right|$, i∈{1, …,s}, r∈{1,…,k}. For each GO term, the quantile or union of neighboring quantiles with the minimal *p*-value is selected. Then the multiple testing correction is applied with the number of tests equal to $s*\overset{k}{\underset{n=2}{\sum }}n$, where ***s*** is the number of all GO terms under study, and ***k*** is the number of quantiles. Every GO term, where the *p*-value passed the multiple testing correction threshold, is considered as fold-change-specific. The fold-change interval with a minimal *p*-value was considered as the magnitude of response.

### 2.3. FSEA Implementation

The FSEA method has been implemented as a FoldGO R package [13] and deposited in the Bioconductor repository (http://bioconductor.org/packages/release/bioc/html/FoldGO.html) along with detailed documentation and examples. The FoldGO R package allows the user to apply both SEA and FSEA to any transcriptome dataset. FoldGO provides the output in both table and chart views depicting the relationship between the fold-change-specific GO terms and specifying their strength of response.

Since the R package requires basic programming knowledge, to make FSEA available for a broader audience, we also implemented it as the web-server FoldGO (https://webfsgor.sysbio.cytogen.ru/). The FoldGO web-server provides a web interface and REST API for remote calculations. The frontend and backend parts of the web-server are implemented using Vue.JS JavaScript and Spring Java frameworks correspondingly. Detailed tutorials for the FoldGO web server and the R package are provided online.

### 2.4. Comparison of FSEA with GSEA and SEA

GSEA and SEA were performed using fgsea [14] and topGO [15] R packages correspondingly with *Homo sapiens* GO annotation (presented in the org.Hs.eg.db R package [16]). GSEA was used with the number of permutations set to 10,000 and fold-change values as a ranking metric. For both SEA and GSEA, the FDR threshold was set to 0.05.

## 3. Results

### 3.1. FSEA Description

We developed the FSEA method to supplement classical GO enrichment analysis (e.g., SEA or GSEA) with the estimate of the magnitude of response for different functional gene groups. FSEA aims to find the relationship between the function of genes and the changes in their expression levels. It is important to highlight that FSEA tests an alternative null hypothesis compared to SEA and GSEA. While SEA and GSEA evaluate if there is a bias in the expression of a functionally-related gene group relative to the whole genome background; FSEA analyzes only differentially expressed genes and assesses if there is a bias in their expression towards a certain range of fold-changes.

**Figure 1:**
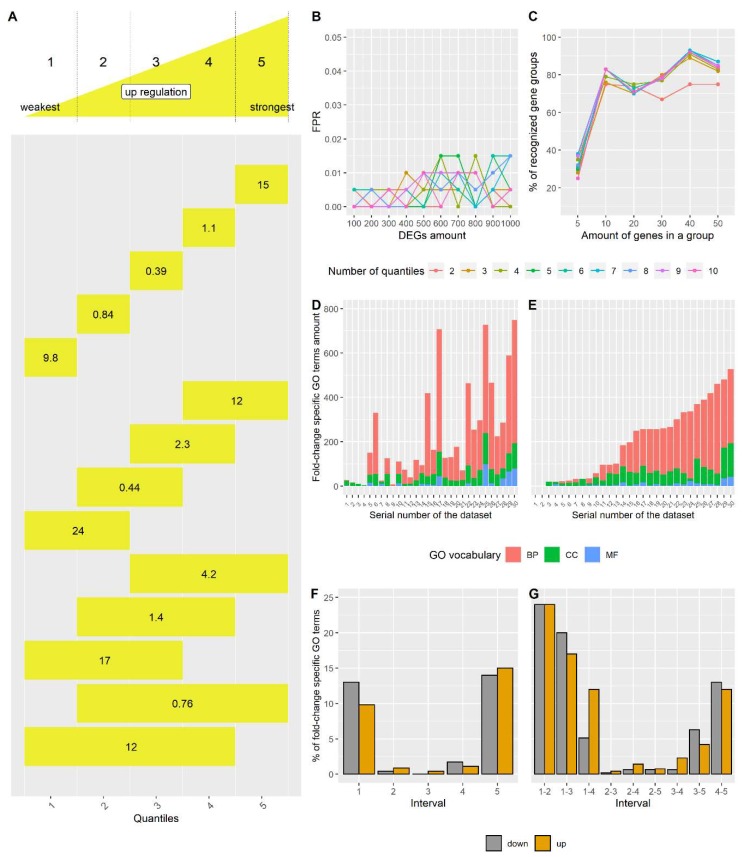
Fold-change-Specific Enrichment Analysis (FSEA) validation. (**A**) Fold-change-specific intervals generated for significantly activated genes (uDEGs, Upregulated Differentially Expressed Genes) in 5-quantile FSEA in the analysis of 30 randomly chosen transcriptome datasets (Table S1). Rectangles denote the fold-change intervals (14 in total). The numbers are the percentages of fold-change-specific Gene Ontology (GO) terms related to the fold-change interval among all fold-change-specific GO terms detected in all intervals. (**B**) Portion of false-positive results. The line chart shows the fraction of false-positive FSEA results (FPR; False Positive Rate) depending on the amount of Differentially Expressed Genes (DEGs) and the number of *n*-quantiles. (**C**) Assessment of FSEA method sensitivity. The line chart shows the percent of correlated gene groups recognized by FSEA depending on the amount of genes in a group and the number of *n*-quantiles. (**D**,**E**). The numbers of fold-change-specific GO terms detected in downregulated DEGs (D) and upregulated DEGs (E) of 30 randomly chosen transcriptome datasets (Table S1). GO terms from three GO vocabularies highlighted by different colors: biological processes (BP, red), cellular components (CC, green) and molecular function (MF, blue). X-axis is the number of the dataset, listed in Supplementary Materials Table S1. (**F**,**G**) The percentage of GO terms defined as fold-change-specific in certain intervals relative to the total number of fold-change-specific GO terms detected in the test datasets (Table S1). The percentages in A and F–G match for upregulated genes.

FSEA utilizes fold-change values from a transcriptome dataset and computes whether DEGs responding within certain fold-change intervals are enriched with particular GO terms. An algorithm behind FSEA consists of two steps (see formal description in Section 2.2). In the first step, FSEA sorts the lists of upregulated and downregulated DEGs (uDEGs and dDEGs) according to their fold-change values (Figure 1A). Then FSEA divides the sorted lists into *n*-quantiles (where *n* is defined by a user) and generates gene sublists for all combinations of neighboring quantiles (hereinafter fold-change intervals). In the second step, FSEA employs gene ontology data for the selected species and estimates for each GO term its enrichment within each fold-change interval compared to the whole uDEGs and dDEGs lists (Table 1). Significance of enrichment is evaluated by Fisher’s exact test with post hoc multiple testing correction procedure (see Section 2.2). GO terms enriched in a specific fold-change interval that passed the multiple testing correction threshold are considered as fold-change-specific and the gene sets annotated to them are regarded as coordinatively regulated with a certain strength by the factor under investigation.

The FSEA method was implemented as the FoldGO Bioconductor R package and as the FoldGO web-server for a set of model species.

### 3.2. FSEA Validation

To assess the adequacy of the FSEA statistical procedures for identification of fold-change-specific GO terms we applied two tests. 

First, we estimated the portion of false-positive results. For this, we took a real dataset [17] for which FSEA identified more than one hundred fold-change-specific GO terms. In order to disrupt the relations between the GO annotation and the genes expression values, the gene identifiers were shuffled to generate 2000 gene expression datasets. FSEA identified just a few fold-change-specific GO terms over all shuffled datasets in total, with most of the shuffled datasets having no significant results. We performed this procedure for different numbers of *n*-quantiles (*n* = 2...10) and varying amounts of DEGs from 100 to 1000 with a step of 100 genes (see Section 2.1.2). As a result, for any *n*-quantile and any quantity of DEGs we observed a fraction of false-positive results less than 2.5% (Figure 1B). This suggests that FSEA is sufficiently reliable to identify fold-change-specific GO terms.

Second, to assess FSEA method sensitivity we sampled 1000 datasets from a multidimensional normal distribution with predefined gene groups of different sizes with high correlation values (see Section 2.1.2). Sensitivity assessment was done by varying the number of *n*-quantiles and the amount of genes in generated groups. FSEA detected more than 75% of groups containing more than 10 genes if their correlation coefficient was above 0.7 (Figure 1C). This analysis showed that FSEA is sensitive enough to detect the functionally-related groups with a coordinated expression behavior.

After validation, we applied FSEA to real datasets for a 5-quantile analysis. As a rule, FSEA gave only a few fold-change-specific GO terms on the datasets with an insufficient number of DEGs (less than 100). Thus, we randomly chose 30 datasets with a representative number of DEGs (>300) from the GEO database. FSEA found fold-change-specific GO terms in all tested datasets (Figure 1D–E). This suggests that fold-change-specific transcriptional response is a universal feature, which therefore should be taken into account in transcriptome analysis. The composition of fold-change-specific GO terms was unique for each dataset. In general, GO terms from “biological processes” GO vocabulary were detected as fold-change-specific more often, while GO terms for “molecular functions” were rare FSEA results (Figure 1D,E). Cellular components were not that abundant but most significant fold-change-specific GO terms (Table S3). These results are logical, as the networks behind cellular components functioning and biological processes regulation have to be more coordinated than the gene sets united by a molecular function. 

We also analyzed which fold-change intervals engage more coordinative response (Figure 1A,F,G). There were two polar attractors—fold-change-specific response was mainly either weak or very strong. Scientists usually pay more attention to the genes with the highest fold changes and to the processes they belong to. FSEA detected many processes activated or repressed weakly and this response was largely understudied. Earlier the importance to consider small changes in RNA expression was highlighted [7] but never studied in depth. 

### 3.3. FSEA Performance on a Cancer-Related Dataset 

To demonstrate FSEA performance, here we discuss functional annotation of one particular dataset, namely, the differential expression data between primary prostate epithelial cell line (HPrEC) and prostate adenocarcinoma cell line (LNCaP), representing essentially healthy and disease states, respectively. For this dataset, we compared the outputs of FSEA (5-quantile) with other widely-used methods for gene set enrichment analysis, SEA, and GSEA [3]. 

**Figure 2:**
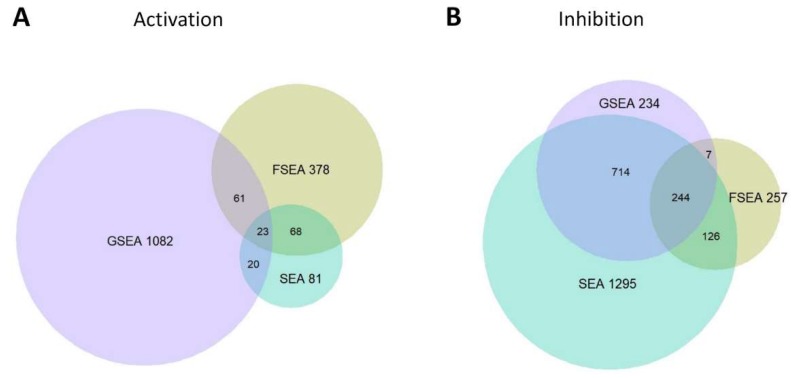
Summary statistics for the Gene Ontology (GO) terms detected by Fold-change-Specific Enrichment Analysis (FSEA), Gene Set Enrichment Analysis (GSEA), and Singular Enrichment Analysis (SEA) in comparison of transcriptomes between HPrEC and LNCaP cell lines (GSE70466). (**A**) Activation of gene expression. (**B**) Inhibition of gene expression.

FSEA results for the cancer-related dataset only partially overlap those detected by GSEA and SEA (Figure 2, Table S2). As mentioned in the Section 3.1, FSEA tests another null hypothesis, so it does not compete but complements GSEA and SEA approaches. Thus, both positive and negative results of FSEA for a particular GO term are valuable, as they suggest if the functionally related gene group responded to a stimulus coordinatively, within specific ranges of fold-changes, or not. Below we discuss three groups of GO terms in more detail: identified by both FSEA and SEA, and either by SEA or FSEA.

#### 3.3.1. FSEA and SEA: FSEA gives an Additional Dimension to SEA Results

**Figure 3:**
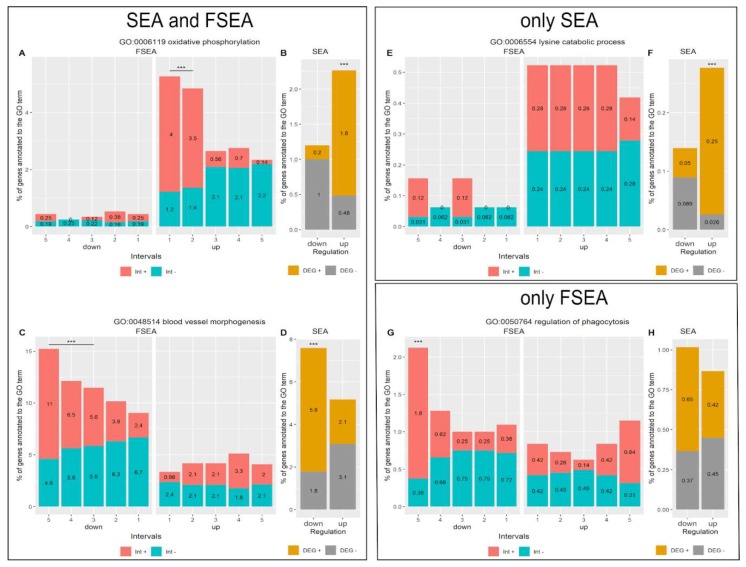
The response pattern of cancer-related processes identified in functional annotation of the LNCaP cell line in comparison to the HPrEC line as overrepresented by either Singular Enrichment Analysis (SEA) (**E**,**F**), or Fold-change-Specific Enrichment Analysis (FSEA) (**G**,**H**), or both (**A–D**). For each Gene Ontology (GO) term, we provide two histograms, A, C, E and G show FSEA output. Red-colored is the percentage of Differentially Expressed Genes (DEGs) related to the GO term and belonging to the fold-change interval compared to all DEGs in this interval; blue-colored is the percentage of DEGs out of the interval but related to the GO term among all DEGs out of the interval. Five-quantile FSEA marks the following intervals: 1—very weak response, 2—weak response, 3—moderate, 4—strong, and 5—very strong. Horizontal lines with asterisks denote the interval on which FSEA shows the significant enrichment of red-colored fractions versus blue ones. B, D, F, and H present the SEA output with (yellow colored) the percentage of DEGs annotated to the GO term in DEGs and (gray colored) the percentage of genes annotated to the GO term in not DEGs. Asterisks denote enrichment of yellow-colored fractions versus gray ones. A,B. Oxidative phosphorylation (GO:0006119). C,D. Blood vessel morphogenesis (GO:0048514). E,F. Lysine catabolic process (GO:0006554). G,H. Regulation of phagocytosis (GO:0050764).

There are 91 GO terms for uDEGs and 370 terms for dDEGs in intersection of FSEA and SEA outputs (Figure 2). These processes are overrepresented in DEGs compared with the whole genome background and enriched in DEGs changing their expression within specific fold-change intervals compared with the whole set of DEGs. The latter means that the functionally related genes alter their expression coordinatively and with a certain magnitude of response. For example, FSEA showed a significant association of the genes related to oxidative phosphorylation (GO:0006119) with a weak activation (Figure 3A). It is known that prostate cancer cells have the shift in metabolism to oxidative phosphorylation [18]. While SEA suggests that this process is influenced by cancerogenesis, FSEA highlights that the genes related to this process are conjugately weakly activated in the LNCaP line.

Another example, the GO term detected by both SEA and FSEA in dDEGs is blood vessel morphogenesis (GO:0048514) (Figure 3C,D). FSEA identified that a notable part of genes associated with this GO term are inhibited from moderate to very strong levels (interval 3–5 out of 5). The Network of Cancer Genes (NCG) [12] identified as oncogenes 56 out of 182 genes, related to this GO term and fold-change-specifically inhibited in the cancer line. Nine of them were tumor suppressors, e.g., *FBXW7* [19] and *BAX* [20,21]. It is known that tumors have abnormal vasculature development [22], FSEA results suggest that to identify the gene networks involved in various aspects of cancerogenesis, relevant genes under strong inhibition should be explored in more detail.

There are many other meaningful fold-change-specific associations detected for this experiment and worth studying by specialists in cancer genomics (Table S2). To sum up, FSEA provides an insight that may help to narrow the set of candidate genes responsible for the observed phenotype by selecting those associated with the fold-change-specific GO term and responding within the significant fold-change interval.

#### 3.3.2. Functional Groups Detected by SEA but Not FSEA: Non-Coordinated Response

Identification of a GO term enrichment by SEA but not by FSEA means that coordination in gene expression was not found for this process. Along with this, we noticed that GO terms identified only by SEA are often presented as a redundant set of nested and mainly general GO terms representing multicomponent processes (Table S2).

The typical profile of expression changes for functionally related gene groups that were detected by SEA but not FSEA is shown in Figure 3E for lysine catabolic process (GO:0006554). Percentages of DEGs annotated to this GO term have approximately uniform distribution across all fold-change intervals. However, this does not interfere with significant enrichment of this process in uDEGs relative to the whole genome background (Figure 3F). Indeed, increased and not coordinated lysine degradation in prostate cancer was shown earlier, as elevated levels of some intermediate metabolites from this process in tumor tissues were detected [23].

Another example of the process related to cancer development that was detected by SEA but not by FSEA is the activation of the coenzyme metabolic process (GO:0006732). The critical role of coenzymes in cancer metabolism is well known [24]. Although FSEA showed that the whole set of genes of this multi-component process is not coordinated in the strength of response, its subgroups can be coordinated fold-change-specifically, e.g., the nested GO term coenzyme biosynthetic process was activated from very weak to moderate levels (Table S2).

#### 3.3.3. Only FSEA: A Quantized Response Invisible for Classical Enrichment Analysis Methods

**Figure 4:**
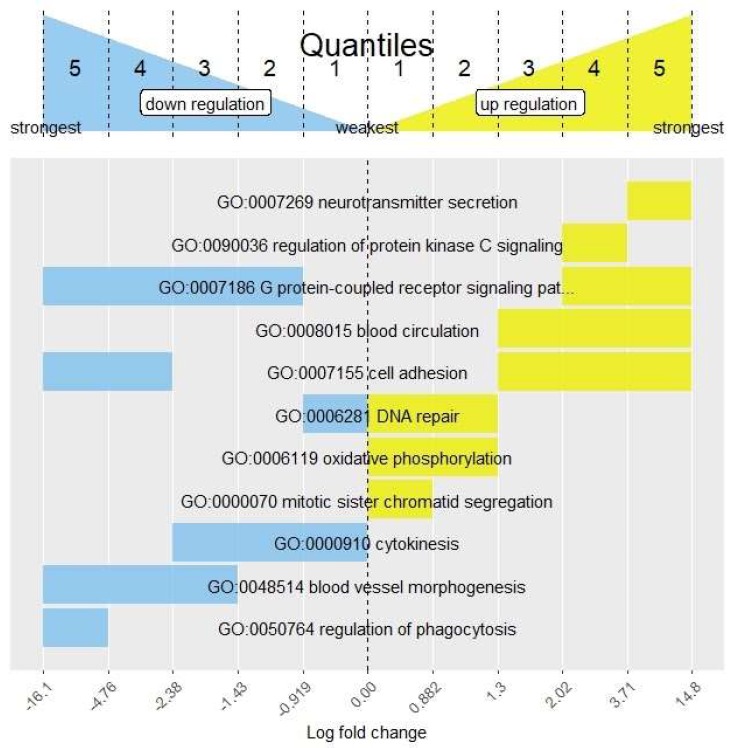
The FoldGO output data for comparison of gene expression between primary prostate epithelial cell line (HPrEC) and prostate adenocarcinoma cell line (LNCaP). The chart provides the fold-change intervals detected by Fold-change-Specific Enrichment Analysis (FSEA) where selected Gene Ontology (GO) terms (the whole list is in Table S2) showed the most significant enrichment compared to the whole Differentially Expressed Genes (DEGs) list. Bars for fold-change-specific GO terms are painted in yellow and blue colors for up- and down-regulated processes, correspondingly.

FSEA detected 439 and 264 GO terms but not SEA for uDEGs and dDEGs, correspondingly (Figure 2) and many of them are cancer-related (Table S2). For example, Figure 4 demonstrates the fold-change-specific intervals for several selected processes. Representative fold-change-specific expression profile is shown in Figure 3G for “regulation of phagocytosis” (GO:0050764). FSEA detected a significant bias towards very strong down-regulation of DEGs associated with this GO term. As the dDEGs related to the process were not enriched, or even depleted, in the rest intervals SEA overlooked this term as associated with dDEGs (Figure 3H). Phagocytosis has long been known as a process closely connected with tumor progression and directly with organism reaction to cancer cells [25] and only FSEA showed the ability to recognize this process as functionally relevant to the LNCaP cell line.

Another example of FSEA, but not a SEA-detected GO term, is neurotransmitter secretion (GO:0007269) that was significantly associated with very strong activation of gene expression (Figure S1). Recently the role of neurotransmitter signaling in tumor progression became an important focus of cancer studies [26]. One of the examples is the *ERG* oncogene that causes overexpression of nicotinic acetylcholine receptors (*nAChRs*) in prostate cancer cells which in turn, under nicotine treatment, induces tumor cell proliferation. These findings show that the processes that are not enriched in the whole DEG sets and overlooked by classical functional annotation, may show specific patterns of gene expression changes and provide an insight into the molecular mechanisms under study.

## 4. Discussion

Here we formalized, validated and demonstrated the potential of Fold-change-Specific Enrichment Analysis (FSEA) for functional annotation of transcriptome data. We developed the FoldGO Bioconductor package and web-server (https://webfsgor.sysbio.cytogen.ru/) to perform functional annotation with both FSEA and SEA. FoldGO can be applied to microarray and RNA-Seq datasets of any species, which has GO annotation.

The conceptual scheme of FSEA has been proposed to study *Arabidopsis thaliana* root transcriptome responded to plant hormone auxin [5]. Here we showed that fold-change-specific response is a rather common phenomenon with a unique composition of fold-change-specific GO terms detected for each individual transcriptome. FSEA analysis of multiple randomly chosen datasets showed that there are two major fold-change-specific responses, either weak or strong. This suggests that the genes with small changes in expression, which are often excluded from the analysis, are not less important than ones with high levels of expression (Figure 1F,G, Table S1).

**Figure 5:**
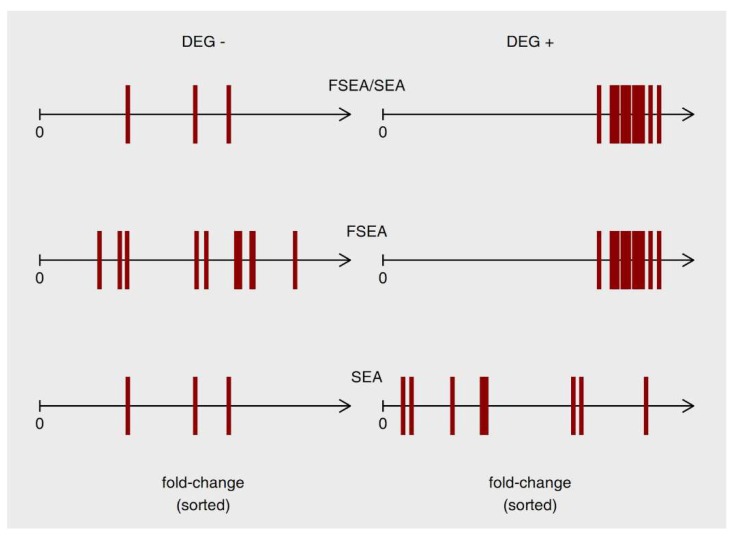
Schematic representation of three functionally-related groups identified as overrepresented in upregulated Differentially Expressed Genes (uDEGs) by Singular Enrichment Analysis (SEA), Fold-change-Specific Enrichment Analysis (FSEA), or both methods. Left and right halves of the plot refer to genes outside and inside uDEGs list, respectively. Genes annotated to the Gene Ontology (GO) term of interest denoted as red vertical bars on the scale of ascendingly sorted fold-change values.

FSEA complements and extends classical methods of functional annotation (SEA and GSEA), providing new and potentially valuable information about coordinated behavior of the functionally-related genes, which resulted in the specific strength of response for this functional group. Figure 5 schematizes the differences between functionally-related gene groups recognized by SEA, FSEA, or by both approaches. These are three different patterns of response and only generalized one has been studied before. In particular, the GO terms that are recognized by FSEA, but not SEA does not show a massive response, but very well coordinated one. 

It is known that tumor subtypes can be defined according to their gene expression profiles [27,28]. Such classification is being used in guidelines for the treatment of early stages of specific cancer types [27]. Since FSEA searches for connectivity between the function and the magnitude of gene expression changes it finds the precisely regulated processes underlying the overall response and forming its frame. Here we discussed FSEA/SEA functional annotation of RNA-Seq experiment on the comparison of gene expression between primary prostate epithelial cell line (HPrEC) and prostate adenocarcinoma cell line (LNCaP). FSEA found more than 1000 GO terms significantly enriched in certain fold-change intervals, and a great part of them were not detected by classical approaches, SEA and GSEA (Figure 2). Among these GO terms, many were firmly related to cancerogenesis, e.g., FSEA found a slight activation in the expression of genes related to mitotic sister chromatid segregation [29] and high levels of activation for the regulation of protein kinase C signaling [30,31] (Figure 4).

Earlier, our approach was tested by a third-party group of researchers on the study of drought stress response in rice [6]. Authors detected differential quantitative regulation of some gene groups under drought stress conditions. They concluded that drought-sensitive rice variety differs from drought-tolerant ones in the compositions of fold-change-specific GO terms lists. Thus, our approach may help to construct the roadmap for converting stress-sensitive varieties to stress-resistant ones by revealing which functional gene sets should be strengthened or inhibited and to what degree.

In addition to gene ontology, one can use for FSEA analysis KEGG pathways [32], BIOGRID protein interactions [33], InterPro domains [34] and other resources of data for functional annotation to reveal more fold-change-specific features associated with DEGs. For example, in Omelyanchuk et al. 2017 [5] we showed the applicability of FSEA method to find the cis-regulatory elements enriched within the promoters of genes having similar fold-changes in transcription. 

## 5. Conclusions

Many tools are available for functional annotation of transcriptome changes. However, these methods either ignore fold-change values or use them only for gene ranking. Here we developed FSEA, the functional annotation method that considers fold-changes in gene expression and by this allows estimating the magnitude of response for biological processes, cellular compartments, and molecular functions. This approach altogether with SEA was implemented in the FoldGO web-server. Comparing FSEA with SEA showed that FSEA provides additional biologically meaningful outcomes to classical functional annotation. FSEA results not only provide a more comprehensive annotation of transcriptome data but also give an insight into the diversity of magnitudes in transcription response for different functional groups.

## Figures and Tables

**Table 1:** The contingency table for Fisher’s exact test to estimate the enrichment of a Gene Ontology (GO) term in a gene set that responds to a stimulus within specific fold-change interval *Q*.

	*Q_r_* +	*Q_r_* −	Total
*GO_i_* +	A	B	A + B
*GO_i_* **−**	C	D	C + D
Total	A + C	B + D	N

## Supplementary Materials

The following are available online at https://www.mdpi.com/2073-4425/11/4/434/s1, Figure S1: Neurotransmitter secretion process (GO:0007269) identified as overrepresented in functional annotation of LNCaP cell line in comparison to HPrEC line by FSEA; Table S1: Metadata on 30 microarray and RNA-Seq datasets from the GEO database [8] selected for FSEA method validation and testing; Table S2: FSEA results for RNA-Seq experiment GSE70466 on comparison of gene expression between primary prostate epithelial cell line (HPrEC) and prostate adenocarcinoma cell line (LNCaP); Table S3: FSEA results for 30 microarray and RNA-Seq datasets from GEO database [8] selected for FSEA method validation; Table S4: Differential expression data for RNA-Seq experiment GSE70466 on comparison of gene expression between primary prostate epithelial cell line (HPrEC) and prostate adenocarcinoma cell line (LNCaP).
