# Supplementary figures and images for: Fold-Change-Specific Enrichment Analysis (FSEA): Quantification of Transcriptional Response Magnitude for Functional Gene Groups
Source: Genes (Basel). 2020 Apr 17;11(4):434. doi: 10.3390/genes11040434 (PMC7230499; doi:10.3390/genes11040434)

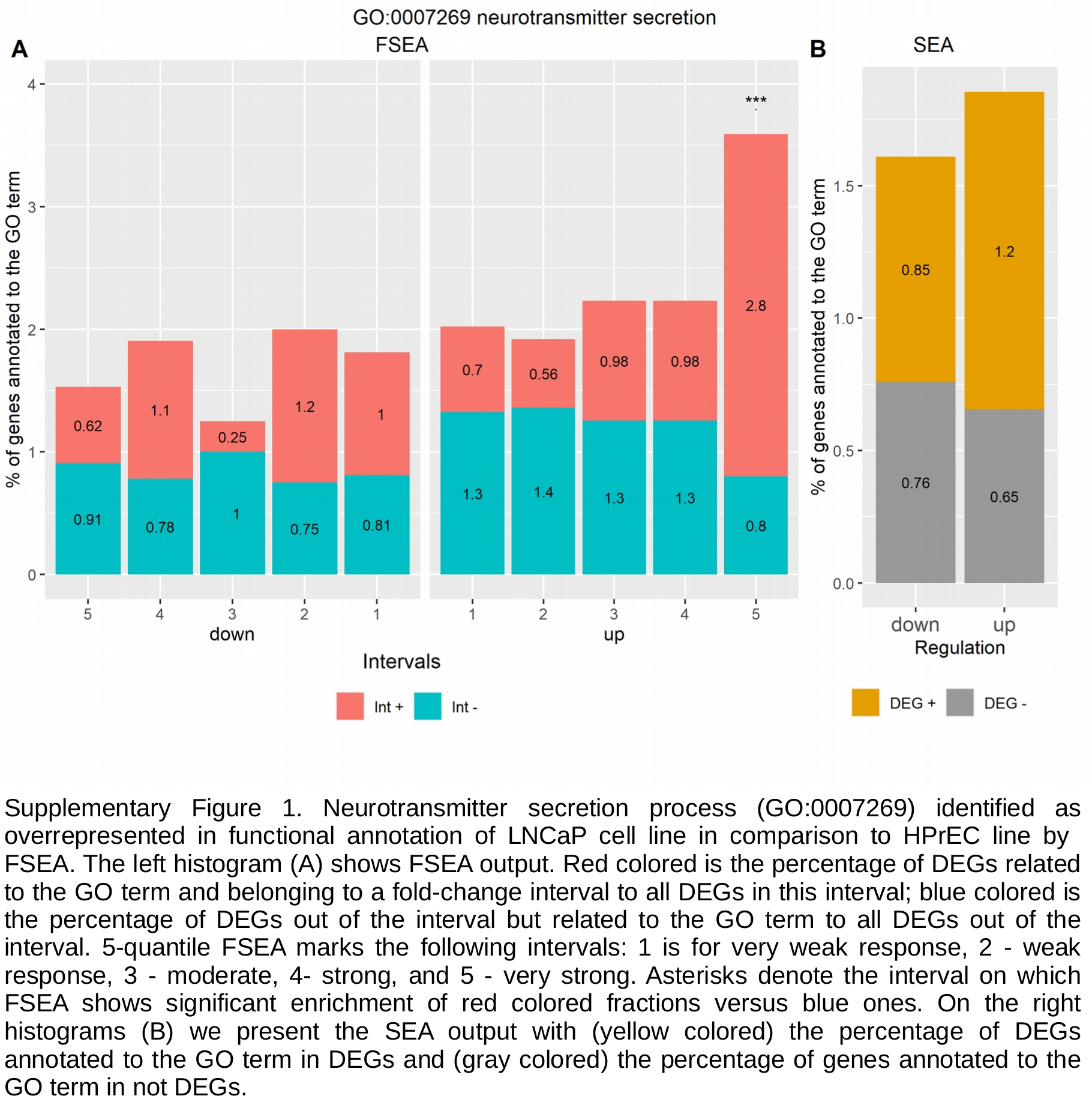

Supplement: Supplementary file 1 [file genes-11-00434-s001.zip › Supplementary Files/Figure S1.png]
